# Performance Analysis of Radial Basis Function Metamodels for Predictive Modelling of Laminated Composites

**DOI:** 10.3390/ma14123306

**Published:** 2021-06-15

**Authors:** Kanak Kalita, Shankar Chakraborty, S Madhu, Manickam Ramachandran, Xiao-Zhi Gao

**Affiliations:** 1 Department of Mechanical Engineering, Vel Tech Rangarajan Dr. Sagunthala R&D Institute of Science and Technology, Avadi 600062, India; 2 Department of Production Engineering, Jadavpur University, Kolkata 737136, India; 3 Department of Automobile Engineering, Saveetha School of Engineering, Saveetha Institute of Medical and Technical Sciences, Chennai 602105, India; madhu.sse@saveetha.com; 4 Data Analytics Lab, REST Labs, Kaveripattinam, Krishnagiri 635112, India; ramachandran.manickam@restlabs.in; 5 School of Computing, University of Eastern Finland, FI-70211 Kuopio, Finland; xiao-zhi.gao@uef.fi

**Keywords:** laminated composites, data-driven model, predictive modeling, metamodel, sampling

## Abstract

High-fidelity structural analysis using numerical techniques, such as finite element method (FEM), has become an essential step in design of laminated composite structures. Despite its high accuracy, the computational intensiveness of FEM is its serious drawback. Once trained properly, the metamodels developed with even a small training set of FEM data can be employed to replace the original FEM model. In this paper, an attempt is put forward to investigate the utility of radial basis function (RBF) metamodels in the predictive modelling of laminated composites. The effectiveness of various RBF basis functions is assessed. The role of problem dimensionality on the RBF metamodels is studied while considering a low-dimensional (2-variable) and a high-dimensional (16-variable) problem. The effect of uniformity of training sample points on the performance of RBF metamodels is also explored while considering three different sampling methods, i.e., random sampling, Latin hypercube sampling and Hammersley sampling. It is observed that relying only on the performance metrics, such as cross-validation error that essentially reuses training samples to assess the performance of the metamodels, may lead to ill-informed decisions. The performance of metamodels should also be assessed based on independent test data. It is further revealed that uniformity in training samples would lead towards better trained metamodels.

## 1. Introduction

Composites are one the most widely used materials of the 21st century. Laminated composite structures have become an inevitable component of modern structural, marine and aerospace applications. Accurate estimation of the static and dynamic performance of such structures is an important task. With modern computing facilities and a plethora of highly accurate numerical strategies and mathematical theories, composite structures are now being extensively analyzed in silico. Despite the high accuracy of numerical approaches, such as the finite element method (FEM), their time-intensiveness often hinders their widespread applications, especially when multiple re-runs are required (e.g., for different sets of ply angles or global optimization tasks). In this regard, metamodels can provide a remarkable saving in computational effort and cost. However, the metamodel by virtue of being an approximate model of the actual one (e.g., FEM, computational fluid dynamics), would lead to some loss in accuracy. The accuracy of a metamodel usually depends on various factors, like type, size and complexity of the problem, training data characteristics, the algorithm used, etc.

Several researchers have already adopted various metamodeling algorithms ranging from polynomial regression (PR) [1,2] to genetic programming (GP) [3] to the artificial neural network (ANN) for estimation of static and dynamic behaviors of laminated composite structures. Applications of the metamodels in laminated structures have also varied from prediction [4,5] to uncertainty quantification [6,7] to single-objective [8,9] and multi-objective optimization [10,11]. Kalita et al. [1] performed a comprehensive study on polynomial regression (PR) metamodels for dynamic analysis of laminated plates. Based on the experiments with classical design of experiments, it was concluded that D-optimal designs would be more suitable as compared to Box–Behnken design and central composite design plans. Further, it was observed that lack of exclusive tests on dedicated test data could lead to ignorance and mistrust on PR metamodels, although many of them had shown excellent prediction on training data but failed miserably on test data. Kalita et al. [3] also carried out a multi-scale optimization of laminated structures using GP metamodels and noticed that GP metamodels could be deployed to efficiently and inexpensively switch between micro to macroscale and vice versa. It was also postulated that as much as 99% of the optimization algorithm running time could be saved by replacing an FE model with a metamodel in a metaheuristic-based global optimization problem of laminated composites. Ganguli [2] developed a mathematical programming methodology based on PR metamodels to design optimal helicopter rotor blades. Dey et al. [12] carried out optimization of composite shells considering the effect of uncertainty. Jafari et al. [13] maximized the fundamental frequency of skew composite plates using a PR metamodel. Heinonen and Pajunen [9] solved a weight minimization problem for stiffened plates using PR and Kriging metamodels. It was concluded that Kriging metamodels would be more advantageous than PR metamodels in single-objective optimization using sequential quadratic programming technique. Todoroki et al. also employed similar PR metamodels coupled with a genetic algorithm to maximize the buckling load of simple composite plates [14], blade-stiffened composite plates [15] and composite shells [16]. It can be revealed from the existing literature that PR metamodels have widely been applied for laminate modelling problems, perhaps due to their easiness of use. However, PR metamodels do not interpolate the sample points and are dependent on the metamodel form selected a priori. Thus, for accurate modeling of laminates where ply angles are considered as the design variables, PR metamodels may not be suitable as the design domain is large (±90°).

Compared to PR metamodels, radial basis function (RBF) metamodels have been less frequently applied for predictive modelling of laminated composites. Rouhi et al. [17] used an RBF metamodel for buckling load optimization of a composite cylinder with variable stiffness. Kaveh et al. [18] solved a similar problem of buckling load optimization to design variable stiffness composite cylinders by conjugating RBF metamodels and Water Strider Algorithm. Nguyen et al. [19] carried out a multi-scale optimization study to reduce the weight of perforated composite structures. They compared the performance of RBF, back-propagation neural network (BPNN) and least square support vector regression (LS-SVM) metamodels by integrating them with an optimizer and reported BPNN to be most efficient. Kalnins et al. [20] compared the performance of RBF, multivariate adaptive regression splines (MARS) and PR for optimization of post-buckling characteristics of damaged composite stiffened structures. Lanzi and Giavotto [21] compared the performance of RBF, ANN and Kriging metamodels in a multi-objective optimization scenario of post-buckling load maximization and weight minimization of composite stiffened panels.

Zhao et al. [22] developed a structural reliability analysis method by incorporating RBF, genetic algorithm and Monte Carlo simulation. They constructed the RBF metamodel from Latin hypercube sampled data. Joy et al. [23] used an ensemble of PR, Kriging and RBF metamodels for the inverse problem of detecting the delamination location by studying the change in natural frequencies. Raturi et al. [24] used an RBF metamodel for stochastic analysis of laminated shells considering the first-ply failure. Similar studies on stochastic assessment of buckling of sandwich panels by incorporating the material and geometric uncertainties in RBF metamodels were carried out by Kumar et al. [25,26]. Dey et al. [27] also contrasted the performance of PR, Kriging, high dimensional model representation, polynomial chaos expansion, ANN, moving least square, support vector regression, MARS, RBF and polynomial neural network metamodels in surrogate modelling of laminated structures. However, it should be noted that in most stochastic modelling studies, the range of design variables is generally very small.

As pointed out by Amouzgar and Stromberg [28], the performance of RBF metamodels had been compared in the past with several other algorithms and their efficacy in predictive modelling of engineering problems had been well established. However, no comprehensive analysis has been carried out on the utility of RBFs in large domain problems. Moreover, the effect of training data uniformity on RBFs has not been studied so far. Thus, in this paper, a comprehensive analysis of RBF metamodels is carried out to ascertain the utility of various basis functions in metamodeling of large domain problems, like laminated structures modelled with respect to ply angles.

The rest of this paper is structured as follows: The objectives of this paper along with the test problems considered are stated in Section 2. Various methods used for sampling, data generation, metamodeling and its evaluation are presented in Section 3. The results of the two test problems are separately discussed in detail in Section 4. Finally, conclusions based on this comprehensive analysis are drawn in Section 5.

## 2. Objectives and Problem Description

**Figure 1:**
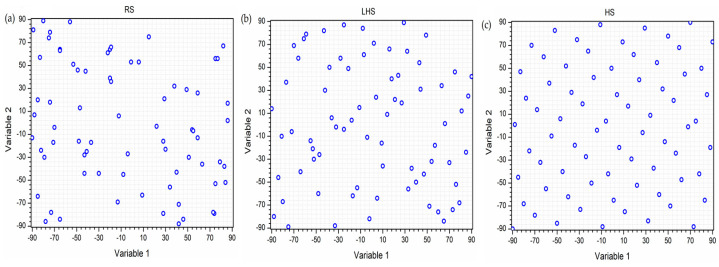
Distribution of two variables of LD-problem (*λ*_1_) in design space for (**a**) RS (**b**) LHS (**c**) HS.

The prime objective of this paper is to comprehensively investigate the viability of RBF metamodels as a reliable surrogate for high-fidelity analysis of laminated composite structures. The second objective is to assess the effect of training data sampling technique on the overall predictive performance of the RBF metamodel. In this regard, three different data sampling techniques, i.e., random sampling (RS), Latin hypercube sampling (LHS) and Hammersley sampling (HS) are considered here. In Figure 1, a comparison of typical sample sets generated by RS, LHS and HS for a typical 2-variable problem is exhibited. It can be noted from this figure that HS has the maximum uniformity in data generation, while RS has the least uniformity in the considered 2-dimensional design space. Since, different performance metrics (like R^2^, leave-one-out cross-validation, *n*-fold cross-validation, mean squared error etc.) have often been proposed by the past researchers for quantification of accuracy of the metamodels, it would be an interesting exercise to investigate the influences of uniformity of the training data on the behaviour of different performance metrics. The third objective is thus set to explore the effects of dimensionality and complexity of the problem on the RBF metamodel’s predictive power. In this direction, two different problems, i.e., a low-dimensional (LD) problem (2 design variables) and a high-dimensional (HD) problem (16 design variables) are considered. For each problem, three different responses (first frequency (*λ*_1_), second frequency (*λ*_2_) and third frequency (*λ*_3_) of laminated composite plates) of varying complexity are taken into account.

### 2.1. Problem 1: Low-Dimensional (LD) Problem 

For the LD problem, a 4-ply symmetric square composite laminate is considered. The ply angles are treated as the design variables with the entire range of possible ply angles (i.e., ±90°) as the design space. However, only discrete values with 1° increment within the ±90° range are considered for training and testing data. The thickness-to-side ratio for the composite plate is taken as 0.005. The boundary condition of the composite plate is assumed to be simply supported on all of its sides. In this paper, graphite-epoxy composite laminates with the following material properties [29] are considered for analysis. 

*E*_1_ = 138 GPa, *E*_2_ = 8.96 GPa, G_12_ = G_13_ = 7.1 GPa, G_23_ = 3.9 GPa, *υ*_12_ = 0.3, *υ*_21_ = 0.0195.

Using a finite element (FE) approach, the first three natural frequencies (*λ*_1_, *λ*_2_ and *λ*_3_) of the composite plates are calculated in the non-dimensional form as $\lambda =\omega a^{2}\sqrt{\rho h/D_{0}}$, where $D_{0}=\frac{E_{2}h^{3}}{12\left(1-\upsilon_{12}\upsilon_{21}\right)}$. Here, *ω*, *a*, *ρ* and *h* represent frequency, side width, density and thickness of the laminate. These three natural frequencies are treated here as the responses. The detailed formulation of the FEM used in this paper can be available in [30,31,32]. The FE formulation adopts a 9-node isoparametric element and is based on first-order shear deformation theory (FSDT).

### 2.2. Problem 2: High-Dimensional (HD) Problem 

In the case of the HD problem, a 32-ply symmetric square composite laminate is considered. Thus, the total number of design variables is 16. The thickness-to-side ratio is taken as 0.04. All the other boundary and geometric conditions are the same as those of the LD problem. 

## 3. Methodology

### 3.1. Sampling Schemes

The application of any metamodeling technique starts with setting up a training dataset. The utility of a metamodel as an accurate and effective predictive tool thus largely depends on how well it has been trained using the given dataset. Hence, the training dataset must adequately represent the underlying features of the design space. Therefore, it makes sense to constitute a training dataset drawn from the design space without any bias. In this paper, three different sampling strategies i.e., RS, LHS and HS are adopted to constitute the training dataset. Sample size also plays an important role in influencing the metamodeling process. Several studies have been conducted in the past to ascertain the best possible sample size. In this paper, the recommendations of Jin et al. [33] are considered to determine the sample size (*n*), which can be expressed as a function of the number of input variables (*p*) and a scaling parameter (*l*) [34].

For LD problem:

*n* = *3l* (*p* + *1*)(*p* + *2*) (1)

For HD problem:

*n* = *l* (*p* + *1*)(*p* + *2*)

Considering *l* = 2, the sample sizes (*n*) for LD and HD problems are considered as 72 and 612 respectively. 

#### 3.1.1. Random Sampling

The RS is one of the most commonly employed sampling strategies because it can generate new sample points without taking into consideration the previously generated sample points. Moreover, the total number of sample points to be generated needs not be specified beforehand. In this paper, *n* sample points are randomly generated between the upper and lower bounds of *p* input variables.

#### 3.1.2. Latin Hypercube Sampling

The LHS, originally introduced by McKay et al. [35], is a statistical technique to generate near-random sample points. A Latin hypercube is analogous to a Latin square in *p*-dimensional space. A Latin square may be defined as an *n* × *n* array that is filled up with *n* different parameter values such that each parameter value occurs only once in each row and in each column. Thus, a Latin hypercube for *n* samples in *p* dimensions is a matrix with *n* rows and *p* columns. In LHS, if the variables are continuous, the number of levels for the variables becomes equal to the total number of samples, thereby each level occurring only once in each dimension. However, in the case of discrete variables, the occurrence of each value is equally possible.

#### 3.1.3. Hammersley Sequence Sampling

The HS [36] is a pseudo-random, low-discrepancy sequence based on the representation of a decimal number in the inverse radix format. The radix values are chosen as the first (*p* − 1) prime numbers. The Hammersley sequence generates a highly uniform sample of *n* data points in *p*-dimensional space. In HS, uniformity in the sample space is maintained in all *p* dimensions, which is not possible to achieve in RS or LHS. However, in HS, it is not necessary that each variable level must occur only once. Some values may occur multiple times while some may be skipped.

### 3.2. Finite Element Method

FEM is a numerical approach for solving various types of engineering problems [30,37,38]. To solve a given problem, it discretizes the problem domain into small parts called elements. Each element is connected with its neighboring elements through nodes.

In this paper, the composite laminates are discretized using a 9-node isoparametric plate bending element. It has been well-established that the choice of plate bending theory has a significant influence on the accuracy of the derived results. Thus, in this paper, FSDT is considered to study the effects of rotary inertia and transverse shear deformation. Due to the paucity of space, the FEM formulation employed in this paper is not explained, but can be found in the previous research works by Kalita et al. [30,31,32]. The detailed FEM formulation is also included in the companion data descriptor to this paper.

**Figure A1:**
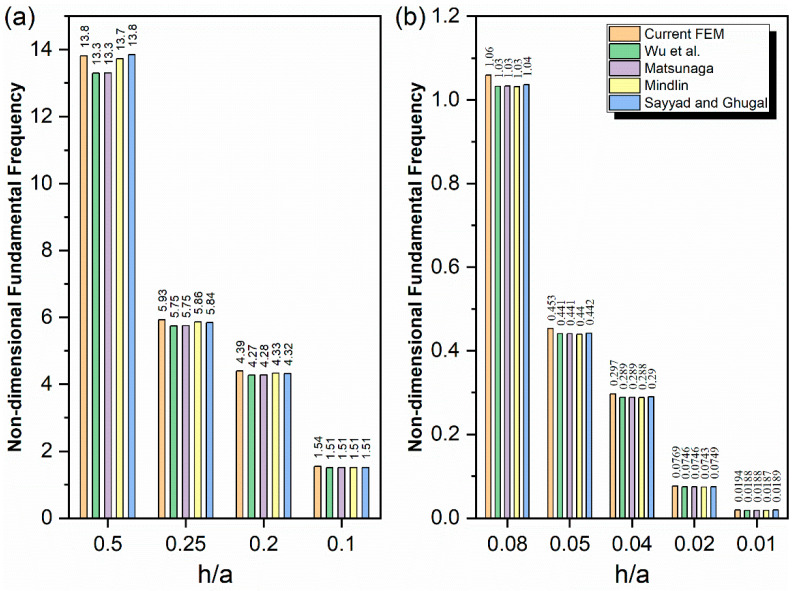
Comparison of non-dimensional fundamental frequencies computed by current FEM and values reported by various researchers [43,44,45,46]. (**a**) moderately thin plates having h/a between 0.5 to 0.1 (**b**) thin plates having h/a between 0.08 to 0.001.

Based on past research works on the same finite element formulation [30,31,32], it is revealed that an 18 × 18 mesh discretization of the composite plate using 9-node isoparametric elements is sufficiently accurate for the considered problems. To demonstrate the accuracy of the current FEM formulation, a validation example is included in Figure A1, Appendix A. It has been exhibited that the application of FSDT can yield results with more than 98% accuracy of the exact solutions and higher-order shear deformation theory-based results. Thus, all the training and testing data employed in this paper are generated based on the aforementioned FE formulation.

### 3.3. Radial Basis Function

The RBFs are commonly used as metamodels in many engineering applications. When provided with a training dataset, it can approximate the underlying model by mapping the outputs as functions of the input variables. It was first applied by Hardy [39] in geophysical research to develop metamodels for topographical contours of geographical data. Since then, it has been applied to diverse problems from almost all domains of engineering [40,41,42]. 

Any general metamodel may be stated as a global approximation function $\tilde{f}\left(x\right)$ developed from a set of given data points $x_{i}\in {ℜ}^{m}$ (*i* = 1, 2, .., *n*) and their corresponding response function $f\left(x\right)$ value. Interpolation of metamodels would generally yield accurate response surfaces while satisfying the following condition:

(2) $$\tilde{f}\left(x\right)=f\left(x_{k}\right),\;k=1,2,...,n$$

Equation (2) indicates that the function *f* and the approximating function $\tilde{f}$ are equal at all the prescribed *n* data points.

An RBF is a function ϕ whose values are real numbers and depend on the distance from the origin, such that $\phi \left(x\right)=\phi \left(\left|\left|x\right|\right|\right)$. The value of RBF may also depend on distance from the center *c*, such that:

(3) $$\phi \left(x,c\right)=\phi \left(\left|\left|x-c\right|\right|\right)$$

where   is any *l_p_* norm. In this paper, *l_2_* norm is considered. 

The generalized metamodel can be expressed as a linear combination of the basis functions across all the data points:

(4) $$\tilde{f}\left(x\right)=\sum_{i=1}^{n}w_{i}\phi \;\left(\left|\left|x-x_{i}\right|\right|\right)$$

where $\tilde{f}\left(x\right)$ can be represented as the sum of *n* radial basis functions, each associated with a different center *x_i_*. Values of the coefficient ($w_{i}$) can be calculated by enforcing the constraints given in Equation (4), to form a linear system of equations:

(5) $$\sum_{i=1}^{n}w_{i}\phi \left(\left|\left|x_{k}-x_{i}\right|\right|\right)=f\left(x_{k}\right),\;k=1,\ldots \;,n$$

Equation (5) can also be expressed in form of matrix as follows:

(6) $$\left[A\right]\left\{w\right\}=\left\{F\right\}$$

where

(7) $$A_{ik}=\phi \left(\left|\left|x_{k}-x_{i}\right|\right|\right),\;i=1,\ldots \;,n;\;k=1,\ldots \;,n$$

(8) $$\left\{w\right\}={\left[w_{1}w_{2}\;\ldots w_{n}\right]}^{T}$$

(9) $$\left\{F\right\}={\left[f\left(x_{1}\right)\;f\left(x_{2}\right)\;\ldots \;f\left(x_{n}\right)\right]}^{T}$$

The above system of equations can be solved to obtain a unique vector {*w*}. This would to an RBF metamodel, as stated in Equation (5), which interpolates all the training data points. Various functional forms of basis function can be adopted to simulate the RBF metamodel.

Linear:

(10) $$\phi_{i}\left(r\right)=r$$

Cubic:

(11) $$\phi_{i}\left(r\right)=r^{3}$$

Gaussian:

(12) $$\phi_{i}\left(r\right)=e^{-{\left(\varepsilon r\right)}^{2}}$$

where *ε* is shape parameter.

Multi-quadratic (MQ):

(13) $$\phi_{i}\left(r\right)=\sqrt{1+{\left(\varepsilon r\right)}^{2}}$$

Inverse multi-quadratic (IMQ):

(14) $$\phi_{i}\left(r\right)=\frac{1}{\sqrt{1+{\left(\varepsilon r\right)}^{2}}}$$

Thin plate spline (TPS):

(15) $$\phi_{i}\left(r\right)=r^{2}\;\ln\left(r\right)$$

In this paper, two different *ε* values (1 and 2) for Gaussian, MQ and IMQ are tried. Hereafter, Gaussian, MQ and IMQ RBFs with *ε* = 2, are referred to as Gauss-2, MQ-2 and IMQ-2, respectively.

## 4. Results and Discussion

### 4.1. Low-Dimensional Problem

**Figure 2:**
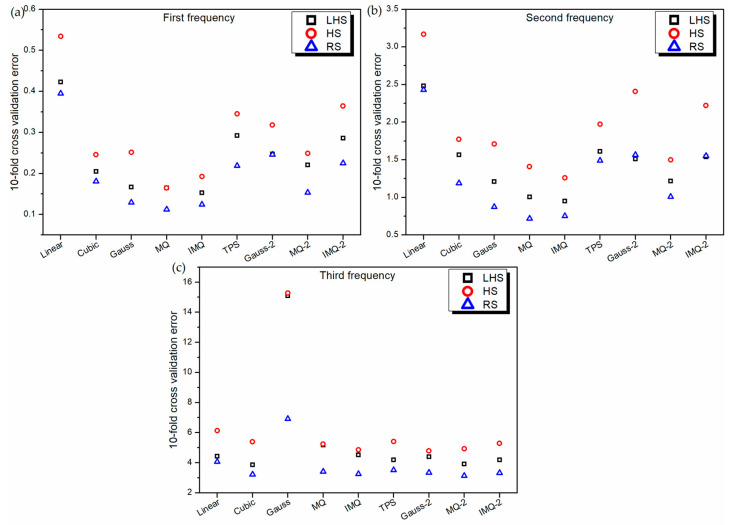
10-fold cross-validation error of various RBF metamodels in LD problem.

The RBF metamodels are now trained using nine different basis functions (linear, cubic, Gaussian, MQ, IMQ, TPS, Gauss-2, MQ-2 and IMQ-2) on three different training datasets (RS, LHS and HS). During the training phase, the 10-fold cross-validation error is estimated while randomly dividing the training dataset into 10 subsets. Figure 2 depicts the best values of 30 trials of 10-fold cross-validation error of various RBF metamodels in the LD problem. It can be revealed that in general, the 10-fold cross-validation error is the least for RBF metamodels trained on the RS dataset and the maximum for the HS dataset. Further, for first frequency and second frequency estimations, MQ-RBF performs the best, while the performance of linear-RBF is the worst. However, in the case of third frequency estimation, MQ-2-RBF performs the best, whereas Gauss-RBF exhibits the maximum 10-fold cross-validation error.

**Figure 3:**
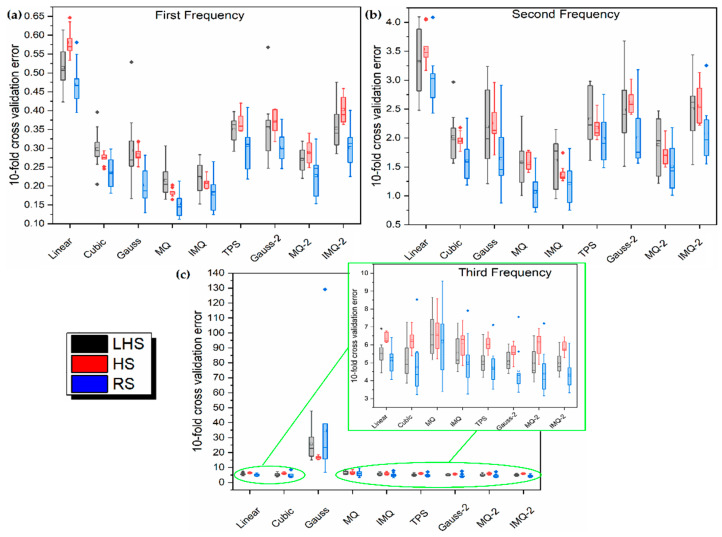
Box plots for 10-fold cross-validation error (30 trials) in LD problem.

To further understand the behavior of the 10-fold cross-validation error in relation to uniformity of the training dataset, 30 independent training datasets, each for RS, LHS and HS, are generated and the 10-fold cross-validation errors are subsequently recorded. Figure 3 exhibits the box plots of the 10-fold cross-validation errors of 30 trials in the LD problem. In general, the RBF metamodels trained on the HS dataset show the least variation. It can be further noted that the overall variability of the 10-fold cross-validation error is the highest for RS. As a whole, MQ-RBF and IMQ-RBF metamodels show the least variability, while Gauss-RBF metamodels have the maximum variability.

**Figure 4:**
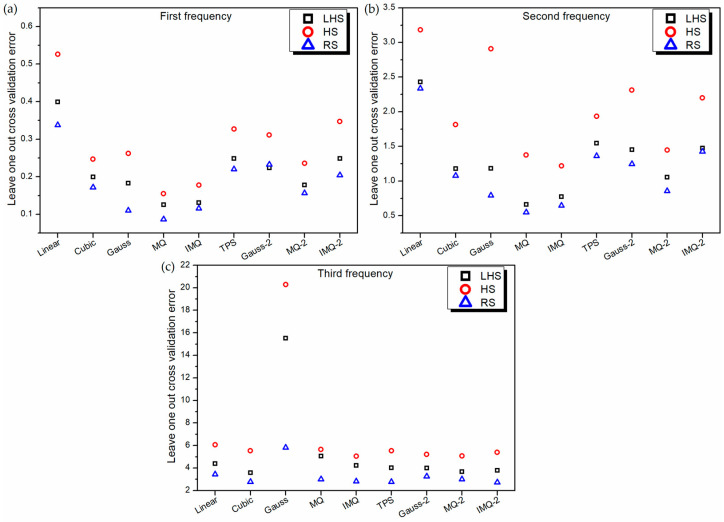
Leave-one-out cross-validation error of various RBF metamodels in LD problem.

The performance of the metamodels is also validated using the leave-one-out cross-validation approach. Because of the similar nature of the leave-one-out cross-validation approach to the 10-fold cross-validation approach, the performance characteristics of various metamodels in Figure 4 are noticed to be similar to those in Figure 2.

Thus, it becomes unveiled from the study of the performance of RBF metamodels that cross-validation approaches (like 10-fold and leave-one-out metrics) essentially reusing training data to assess the model accuracy would in general, show better performance when trained on randomly drawn (RS) datasets. This is because of the inherent mechanism of the cross-validation approaches, which randomly select and segregate some portion of the training data to later act as a validation subset. However, in sampling techniques, like LHS and HS, the overall training dataset is so selected that it is (as much as possible) uniformly spread over the entire design space to be modeled. Therefore, withholding some data (for calculation of cross-validation errors) from these training datasets would have detrimental effects on the training and subsequently on the performance of the metamodel. Thus, any typical sample point of the HS dataset has more influence on the overall predictive capability of the metamodel as compared to a typical sample point of the RS dataset. This is why it can be noticed that as the uniformity of the training dataset increases, the performance of metamodels on cross-validation approaches drops.

**Figure 5:**
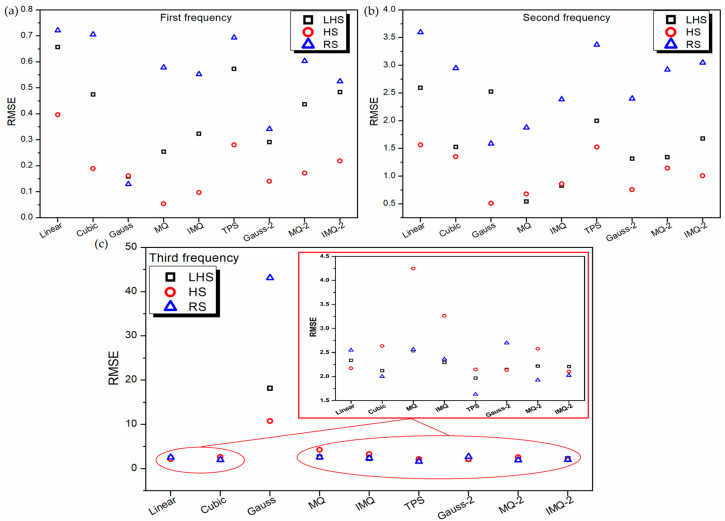
RMSE values of various RBF metamodels in LD problem for testing data.

**Figure 6:**
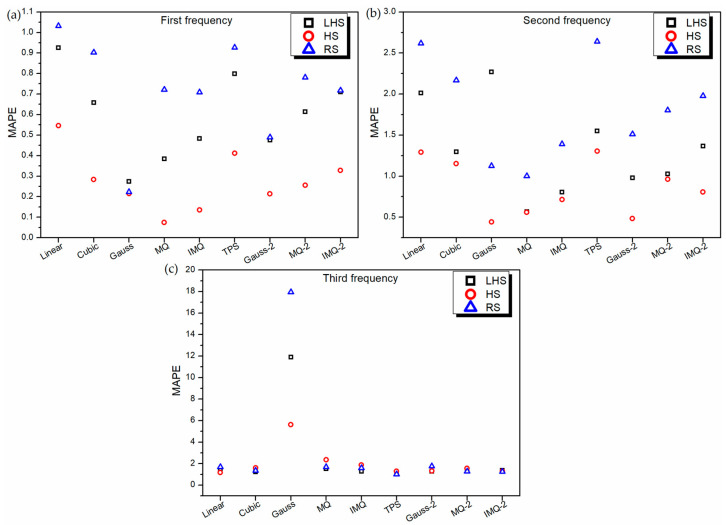
MAPE values of various RBF metamodels in LD problem for testing data.

To further assess the performance of RBF metamodels, a 20-sample point random sampling test dataset is generated using FEM formulation. The performance of all the metamodels is validated based on this test dataset using root mean squared error (RMSE) and mean absolute percentage error (MAPE) as the metrics, as portrayed in Figure 5 and Figure 6 respectively. It can be noticed from Figure 5, for all the RBF metamodels, the value RMSE is the least for HS data in the case of first and second frequency estimations. However, in the case of third frequency estimation, in general, the RSME value of all the RBF metamodels trained on RS data is the least. It is also interesting to note that similar to the 10-fold cross-validation error plot (Figure 2c) and leave-one-out cross-validation plot (Figure 4c), the values of RMSE (Figure 5c) and MAPE (Figure 6c) of Gaussian-RBF metamodels are unusually high for third frequency estimation.

**Figure 7:**
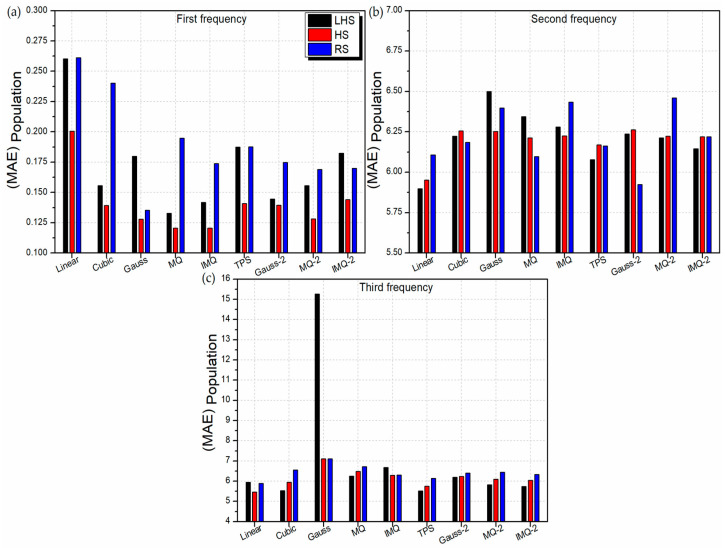
MAE values of various RBF metamodels in LD problem considering entire population.

**Figure 8:**
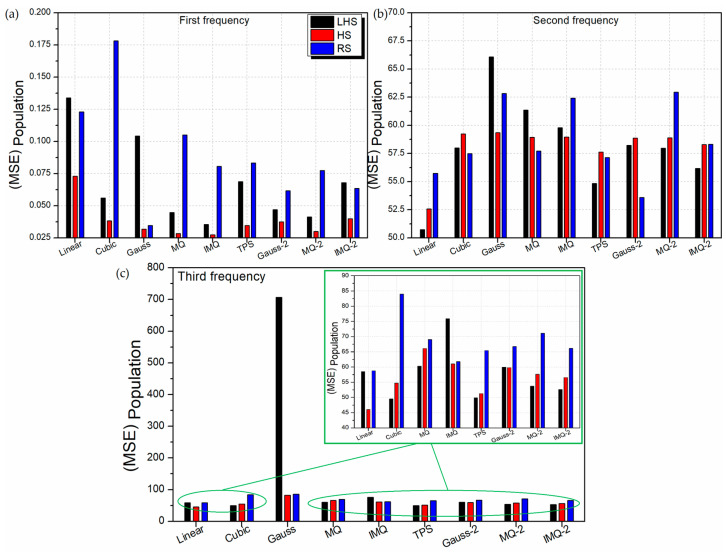
MSE values of various RBF metamodels in LD problem considering the entire population.

Further, the performance of the RBF metamodels is assessed based on the entire population of the 2-variable LD problem. For this, 32,761 sample points are generated considering the LD problem of 4-ply symmetric laminate design with discrete angles of 1°. The performance of the RBF metamodels with respect to mean absolute error (MAE) and mean squared error (MSE) values for the entire population is depicted in Figure 7 and Figure 8 respectively. From Figure 7a and Figure 8a, it can be observed that the HS trained RBF metamodels comprehensively outperform the LHS and RS trained RBFs for first frequency estimation.

**Figure A2:**
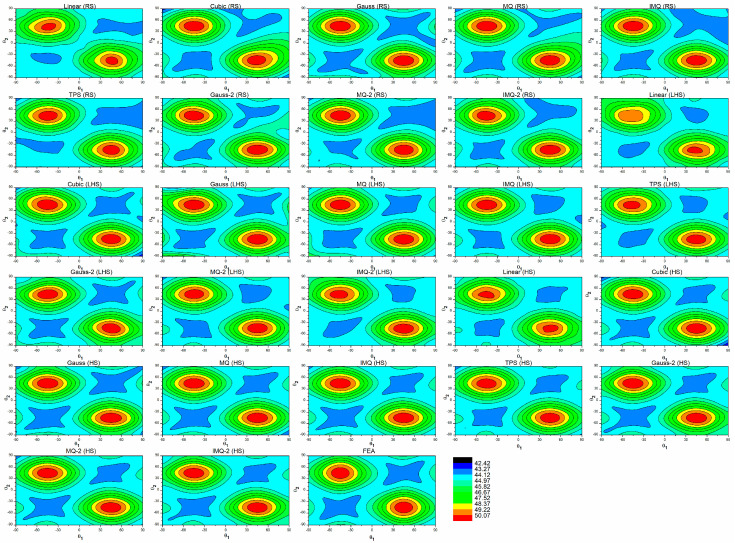
2D contour plots for first frequency of the LD problem generated by various RBF metamodels.

Among different basis functions, MQ and IMQ show the most promising results for first frequency estimation. For second frequency estimation (Figure 7b and Figure 8b), the linear-RBF metamodel trained on the LHS dataset has the least error. In case of third frequency estimation (Figure 7c and Figure 8c), linear-RBF metamodel trained on the HS dataset records the lowest deviation from the true values. The performance of the metamodels with respect to different error metrics is presented in Appendix B through Table A1, Table A2 and Table A3. The 2D contour plots depicting the prediction performance of various RBFs trained with different datasets for first frequency estimation are shown in Figure A2 along with the corresponding FEM solutions. It is clear that in general, the HS trained RBFs have better encapsulated the intricacies in the design space. The 2D contours of HS-trained MQ and IMQ RBFs are observed to be almost identical to the FEM solutions.

### 4.2. High-Dimensional Problem

**Figure 9:**
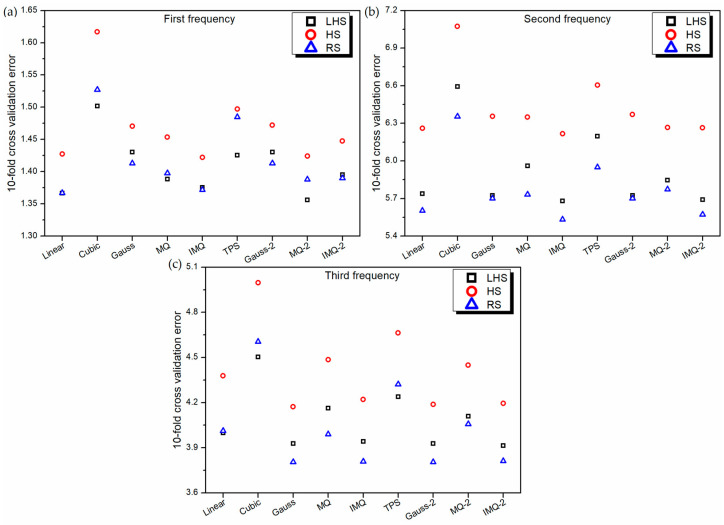
10-fold cross-validation error of various RBF metamodels in HD-problem.

**Figure 10:**
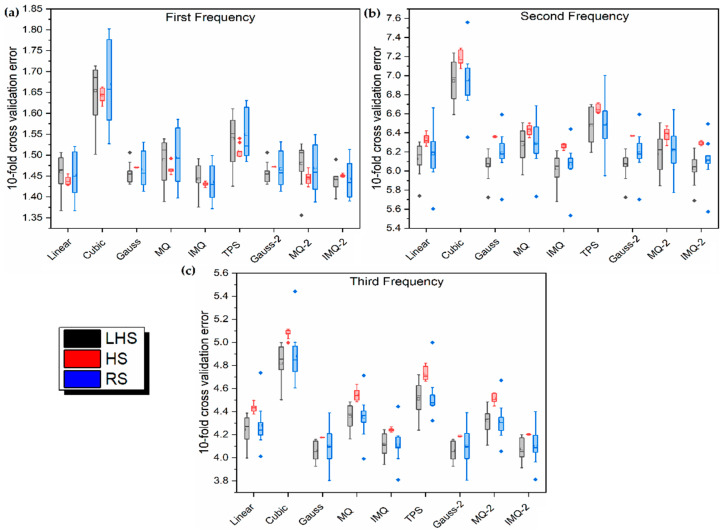
Box plots for 10-fold cross-validation error in HD problem.

The HD problem, as indicated earlier, is a 16-design variable problem, where the variables are the ply angles of 16 laminas of the composite plate with each having a design bound of ±90°. Figure 9 depicts the 10-fold cross-validation error for different RBF metamodels trained on various datasets. It can be observed that the 10-fold cross-validation error is the minimum for the RS dataset and maximum for the HS dataset. Thus, as expected, higher uniformity in the spread of sample points in a training dataset imparts each sample point with more influence on the training goal as compared to a typical sample point from a less uniform dataset. However, it is interesting to note that the variability of 10-fold cross-validation errors on 30 independent trials (Figure 10) is the lowest for the HS dataset and highest for the RS dataset. Thus, when compared for all solutions, the worst solution may also belong to the RS dataset. The negligible variability of 10-fold cross-validation errors for HS datasets makes them more attractive for such HD problems since any typical HS training solution is more reliable and thereby would not require a large number of independent trials (e.g., 30 trials used in this paper) to ascertain the accuracy of the solutions. In real-world situations, a smaller number of trials directly corresponds to less computational cost and quick model deployment time.

**Figure 11:**
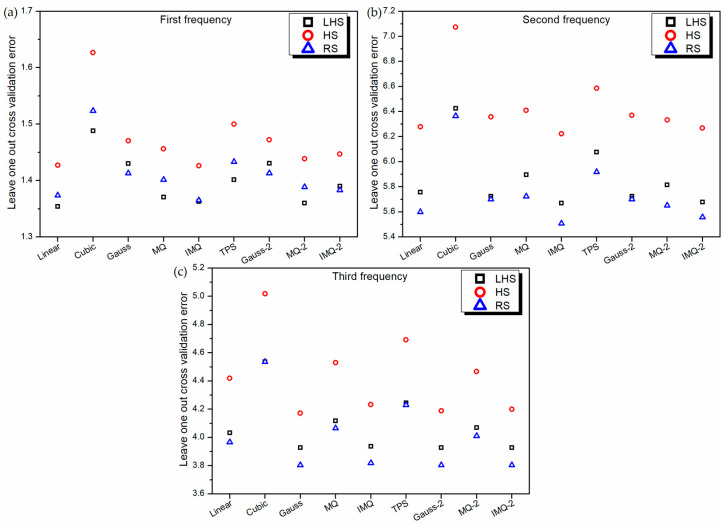
Leave-one-out cross-validation error of various RBF metamodels in HD-problem.

The leave-one-out cross-validation error of the RBF metamodels in the HD problem is presented in Figure 11. For first frequency RBF metamodels, the lowest leave-one-out cross-validation error is noticed for the LHS dataset, whereas, for second and third frequency modelling, the leave-one-out cross-validation error is the lowest for the RS dataset. The MQ (LHS), IMQ (RS) and IMQ-2 (RS) are the best performing RBF metamodels based on the leave-one-out cross-validation error for the HD problem of first, second and third frequency modelling respectively.

**Figure 12:**
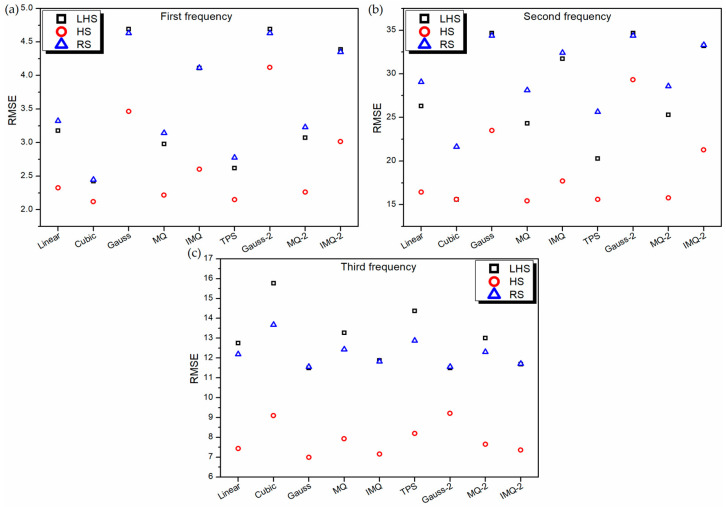
RMSE of various RBF metamodels in HD problem for testing data.

**Figure 13:**
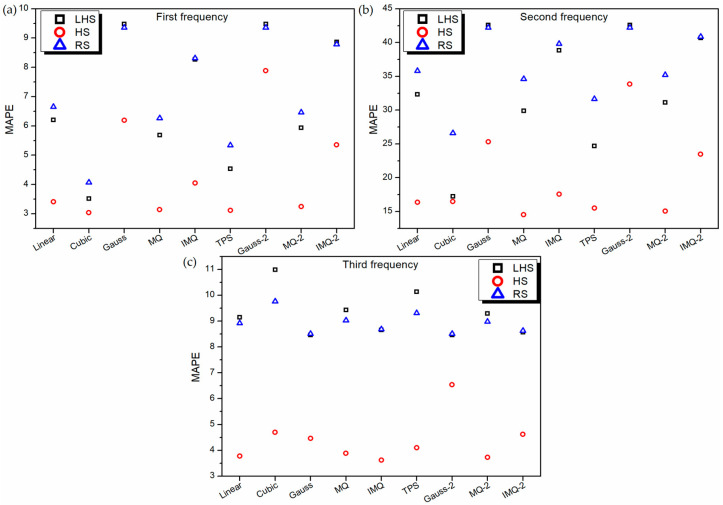
MAPE of various RBF metamodels in HD problem for testing data.

An independent test dataset of 50 random samples is again generated and the accuracy of the RBF metamodels is assessed based on RMSE and MAPE values, as shown in Figure 12 and Figure 13 respectively. Cubic (HS) emerges out as the best RBF metamodel for first frequency modelling. Based on the 10-fold cross-validation error and leave-one-out cross-validation error as shown in Figure 9 and Figure 11, respectively, cubic (HS) RBF is the worst metamodel for modelling of all the frequencies in the considered HD problem. However, it should be noted that in absolute terms of the cross-validation error, cubic (HS) RBF is only 19.5%, 28% and 31.5% worst as compared to the best metamodel for first, second and third frequencies. Contrary to this, the worst metamodel in absolute terms of MAPE on testing dataset is 212% (Gauss-2 (LHS)), 193% (Gauss-2 (LHS)) and 203% (cubic (LHS)) worst as compared to the best metamodel for first (cubic (HS)), second (MQ (HS)) and third (IMQ (HS)) frequencies. Further, from Figure 12 and Figure 13, it can be noticed that irrespective of the basis function adopted, RBF metamodels trained on HS datasets are significantly better than the metamodels trained on LHS and RS datasets. 

In case of the LD problem, it is unveiled that MQ basis functions perform well in all the examples, while for the HD problem, it is difficult to choose the best RBF metamodel. Therefore, a multi-criteria decision-making approach in the form of TOPSIS is employed here to evaluate and rank various RBF metamodels for all the examples. The rankings of the metamodels using TOPSIS are presented in Table A4. 

## 5. Conclusions

In this paper, a comprehensive analysis is carried out on the utility of RBF metamodels in the predictive modelling of laminated composites while considering nine different basis functions. The effect of problem dimension is analyzed using two different problems, i.e., an LD problem of 2 design variables and an HD problem of 16 design variables. The ply angles of each lamina with a design bound of ±90° are treated as the design variables. Further, the role of uniformity of the training data on the predictive ability of RBF metamodels is also studied in detail while considering three different sampling strategies, i.e., RS, LHS and HS. Based on the extensive investigations on the FEM generated high-fidelity data, the following conclusions can be drawn:

(a) The RBF metamodels trained on RS datasets have the best 10-fold cross-validation error and leave-one-out cross-validation error. However, this excellent prediction on training data does not necessarily correspond to excellent prediction (in terms of MAPE and RMSE) on independent test data. In fact, in all the three responses of LD problem, the worst MAPE and RMSE values are recorded for RBFs trained on the RS dataset.
(b) The RBF metamodels trained on HS datasets have the best prediction with respect to MAPE and RMSE on independent test data. However, for all the three responses of both LD and HD problems, HS-data-trained RBFs show the worst 10-fold cross-validation error and leave-one-out cross-validation error. Nevertheless, in case of the LD problem, for the best (in terms of MAPE and RMSE) HS-data-trained RBF metamodels, the 10-fold cross-validation error is 47% (first frequency), 138% (second frequency) and 95% (third frequency) worst as compared to the overall best RBF metamodels. In case of the HD problem, these deviations are much lower, i.e., 19% (first frequency), 15% (second frequency) and 11% (third frequency). Thus, despite using metrics, like 10-fold cross-validation error and leave-one-out cross-validation error, performance measurement of metamodels on independent test data should be encouraged.
(c) In general, irrespective of the sampling strategy and basis function, all RBF metamodels show better performance on the HD problem as compared to the corresponding metamodels for the LD problem. It should be noted that in terms of design variables, the HD problem is 8 times more complex than the LD problem, whereas the training datasets used have a ratio of 8.5:1 for HD and LD problems. Thus, the size of the training dataset has more influence on the metamodel’s predictive performance as compared to the number of variables.
(d) Using TOPSIS, it can be observed that in general, MQ basis functions perform well for LD problem, whereas, for HD-problem, linear and MQ basis functions perform with high reliability.

Thus, it can be concluded that RBF metamodels can be employed to accurately estimate the frequency parameters of laminated composite structures. Further, for large-dimensional complex problems, low discrepancy sampling methods, like Hammersley is likely to be more effective in developing global metamodels. The scope of this paper may include studying the effect of size of the training datasets as well as to apply other metamodeling approaches, like Kriging, support vector regression etc. for predictive modeling of laminated composite structures. 

## Data Availability

The data presented in this study are available in the article.

## Appendix A. Validation of the FEM Formulation

To demonstrate the accuracy of the FEM formulation used in the current paper, an example from literature is solved and compared with published results by various researchers. An all sides simply supported symmetric four layered cross-ply laminated composite square plate is considered. The material properties are $\frac{E_{1}}{E_{2}}=40,\;\frac{E_{3}}{E_{2}}=1,\;\frac{G_{12}}{E_{2}}=\frac{G_{13}}{E_{2}}=0.6\;,\frac{G_{23}}{E_{2}}=0.5\;and\;\vartheta_{12}=\vartheta_{13}=\vartheta_{23}=0.25$. The thickness-to-length ratios (h/a) are varied between 0.5 to 0.001. The current FEM results are compared with those of Wu et al. [43], Matsunaga [44], Mindlin [45], Sayyad and Ghugal [46]. While the current work uses FSDT, Wu et al. [43] used a local higher order shear deformation theory (HSDT), Matsunaga [44] used a global HSDT, Mindlin [45] pioneered the FSDT and Sayyad and Ghugal [46] used a trigonometric shear deformation theory (TSDT). Theoretically, HSDTs and TSDT are more accurate than the FSDT. However, in context of this study the current results are seen to be at par with the HSDTs and TSDT. This is because the considered plates in the example are thin or moderately thin plates, on which the effect of transverse shear strain is negligible.

## Appendix B. Predictive Performance of the RBF Metamodels

**Table A1:** Comparison of all metamodels based on various metrics for first frequency.

Metamodel	LD Problem					HD Problem				
	L_10_O CV ^1^	LOOCV ^2^	MAE	MAPE	MSE	L_10_O CV	LOOCV	MAE	MAPE	MSE
Linear (LHS)	0.4230	0.3996	0.4201	0.9253	0.4310	1.3667	**1.3542** ^3^	2.7372	6.2076	10.0952
Cubic (LHS)	0.2050	0.1997	0.2988	0.6584	0.2251	1.5018	1.4881	1.5735	3.5190	5.8822
Gauss (LHS)	0.1669	0.1830	0.1231	0.2742	0.0252	1.4303	1.4303	4.1653	9.4792	22.0251
MQ (LHS)	0.1646	0.1256	0.1739	0.3847	0.0647	1.3886	1.3705	2.5087	5.6825	8.8845
IMQ (LHS)	0.1527	0.1308	0.2188	0.4833	0.1045	1.3754	1.3630	3.6361	8.2695	16.8776
TPS (LHS)	0.2921	0.2488	0.3628	0.7991	0.3278	1.4256	1.4016	2.0118	4.5406	6.8539
Gauss-2 (LHS)	0.2469	0.2234	0.2144	0.4752	0.0848	1.4304	1.4304	4.1655	9.4794	22.0243
MQ-2 (LHS)	0.2205	0.1784	0.2783	0.6133	0.1909	**1.3562**	1.3602	2.6208	5.9402	9.4500
IMQ-2 (LHS)	0.2858	0.2483	0.3220	0.7104	0.2343	1.3953	1.3899	3.8974	8.8659	19.2459
Linear (HS)	0.5337	0.5264	0.2478	0.5454	0.1575	1.4274	1.4272	1.5109	3.4106	5.4063
Cubic (HS)	0.2455	0.2470	0.1286	0.2837	0.0357	1.6169	1.6264	**1.3604**	**3.0373**	**4.4954**
Gauss (HS)	0.2513	0.2619	0.0967	0.2146	0.0261	1.4704	1.4706	2.7284	6.1930	12.0072
MQ (HS)	0.1644	0.1552	**0.0338**	**0.0748**	**0.0029**	1.4535	1.4562	1.3976	3.1444	4.9146
IMQ (HS)	0.1921	0.1775	0.0616	0.1358	0.0094	1.4221	1.4262	1.7868	4.0455	6.7669
TPS (HS)	0.3453	0.3271	0.1868	0.4119	0.0789	1.4970	1.4997	1.3861	3.1114	4.6152
Gauss-2 (HS)	0.3176	0.3113	0.0966	0.2133	0.0196	1.4721	1.4721	3.4685	7.8823	16.9645
MQ-2 (HS)	0.2487	0.2355	0.1157	0.2552	0.0295	1.4241	1.4386	1.4404	3.2457	5.1219
IMQ-2 (HS)	0.3640	0.3468	0.1484	0.3274	0.0478	1.4473	1.4469	2.3586	5.3527	9.0770
Linear (RS)	0.3947	0.3380	0.4685	1.0320	0.5203	1.3672	1.3736	2.9342	6.6511	11.0335
Cubic (RS)	0.1809	0.1720	0.4091	0.9032	0.4979	1.5272	1.5237	1.8202	4.0734	5.9839
Gauss (RS)	0.1291	0.1102	0.1005	0.2232	0.0168	1.4132	1.4132	4.1115	9.3557	21.4504
MQ (RS)	**0.1120**	**0.0865**	0.3262	0.7212	0.3351	1.3976	1.4015	2.7707	6.2682	9.8799
IMQ (RS)	0.1245	0.1156	0.3207	0.7084	0.3051	1.3718	1.3652	3.6583	8.3158	16.9313
TPS (RS)	0.2187	0.2203	0.4209	0.9268	0.4807	1.4848	1.4335	2.3665	5.3376	7.7091
Gauss-2 (RS)	0.2463	0.2325	0.2214	0.4889	0.1167	1.4132	1.4132	4.1114	9.3555	21.4514
MQ-2 (RS)	0.1531	0.1568	0.3540	0.7813	0.3638	1.3880	1.3887	2.8525	6.4599	10.4330
IMQ-2 (RS)	0.2253	0.2040	0.3258	0.7181	0.2761	1.3900	1.3831	3.8646	8.7897	18.9036

^1^ 10-fold cross validation error, ^2^ Leave one out cross validation error, ^3^ Bold face indicates best value column-wise.

**Table A2:** Comparison of all metamodels based on various metrics for second frequency.

Metamodel	LD Problem					HD Problem				
	L_10_O CV	LOOCV	MAE	MAPE	MSE	L_10_O CV	LOOCV	MAE	MAPE	MSE
Linear (LHS)	2.4798	2.4290	1.6727	2.0130	6.7348	5.7387	5.7572	23.1989	32.3538	691.9223
Cubic (LHS)	1.5656	1.1782	1.0318	1.2958	2.3316	6.5919	6.4240	12.8804	17.2615	243.1132
Gauss (LHS)	1.2103	1.1837	1.8135	2.2702	3898	5.7234	5.7233	30.4541	42.5952	1202.6393
MQ (LHS)	1.0065	0.6612	0.4568	0.5686	0.2936	5.9608	5.8960	21.4918	29.9017	590.7010
IMQ (LHS)	0.9518	0.7734	0.6413	0.8045	0.6841	5.6802	5.6711	27.7195	38.8525	1006.2652
TPS (LHS)	1.6116	1.5472	1.2711	1.5495	4.0019	6.1969	6.0759	17.9242	24.6904	411.5608
Gauss-2 (LHS)	1.5109	1.4530	0.7466	0.9779	1.7330	5.7239	5.7239	30.4567	42.5989	1202.8600
MQ-2 (LHS)	1.2158	1.0548	0.8331	1.0279	1.7965	5.8464	5.8157	22.3485	31.1340	640.3793
IMQ-2 (LHS)	1.5388	1.4738	1.0932	1.3653	2.8164	5.6904	5.6790	29.0572	40.6952	1101.3604
Linear (HS)	3.1686	3.1799	1.0593	1.2912	2.4418	6.2596	6.2791	12.2203	16.3708	270.1005
Cubic (HS)	1.7698	1.8154	0.8917	1.1526	1.8309	7.0733	7.0739	12.4824	16.4916	243.5307
Gauss (HS)	1.7095	2.9085	**0.3440**	**0.4432**	**0.2594**	6.3550	6.3566	18.7299	25.3252	552.5873
MQ (HS)	1.4067	1.3765	0.4381	0.5602	0.4624	6.3493	6.4100	**10.9620**	**14.5482**	**237.6996**
IMQ (HS)	1.2609	1.2194	0.5584	0.7144	0.7371	6.2169	6.2224	13.1118	17.5664	313.6982
TPS (HS)	1.9730	1.9347	1.0212	1.3030	2.3211	6.6053	6.5867	11.6785	15.5172	243.9042
Gauss-2 (HS)	2.4066	2.3104	0.3956	0.4845	0.5706	6.3696	6.3696	24.5857	33.8412	859.1787
MQ-2 (HS)	1.4986	1.4456	0.7525	0.9632	1.3083	6.2661	6.3321	11.3002	15.0440	249.1558
IMQ-2 (HS)	2.2214	2.1980	0.6456	0.8079	1.0117	6.2652	6.2675	17.2876	23.4843	452.8401
Linear (RS)	2.4286	2.3346	2.2050	2.6185	12.9425	5.6040	5.6002	25.6846	35.8118	845.2555
Cubic (RS)	1.1874	1.0802	1.7935	2.1677	8.6909	6.3547	6.3643	19.3016	26.5988	468.7819
Gauss (RS)	0.8751	0.7927	0.9232	1.1250	2.5222	5.7013	5.7013	30.1709	42.2127	1182.6153
MQ (RS)	**0.7173**	**0.5487**	0.8503	1.0024	3.5183	5.7319	5.7249	24.8646	34.6374	790.1790
IMQ (RS)	0.7523	0.6464	1.1646	1.3907	5.6984	**5.5327**	**5.5092**	28.4928	39.8291	1051.2330
TPS (RS)	1.4883	1.3626	2.1791	2.6399	11.3841	5.9504	5.9193	22.7879	31.6445	658.0262
Gauss-2 (RS)	1.5656	1.2460	1.2379	1.5113	5.7580	5.7018	5.7018	30.1719	42.2150	1182.8340
MQ-2 (RS)	1.0100	0.8575	1.5162	1.8034	8.5495	5.7739	5.6515	25.2775	35.2279	817.4594
IMQ-2 (RS)	1.5527	1.4264	1.6484	1.9778	9.3007	5.5730	5.5584	29.2269	40.8789	1108.6229

**Table A3:** Comparison of all metamodels based on various metrics for third frequency.

Metamodel	LD Problem					HD Problem				
	L_10_O CV	LOOCV	MAE	MAPE	MSE	L_10_O CV	LOOCV	MAE	MAPE	MSE
Linear (LHS)	4.4359	4.3846	1.8338	1.4141	5.4576	3.9984	4.0324	10.5484	9.1509	162.5176
Cubic (LHS)	3.8595	3.5830	1.6243	1.2218	4.5104	4.5029	4.5363	12.5952	10.9869	248.5740
Gauss (LHS)	15.098	15.524	15.132	11.9124	328.71	3.9279	3.9279	9.8104	8.4642	132.2075
MQ (LHS)	5.1684	5.0520	2.0034	1.5251	6.4269	4.1635	4.1168	10.8483	9.4278	176.0082
IMQ (LHS)	4.5200	4.2339	1.7250	1.2837	5.2536	3.9417	3.9364	10.0116	8.6562	141.2009
TPS (LHS)	4.1874	4.0146	1.4996	1.1313	3.8723	4.2397	4.2453	11.6407	10.1345	206.4641
Gauss-2 (LHS)	4.3990	3.9991	1.6313	1.2787	4.5993	3.9280	3.9280	9.8088	8.4627	132.1455
MQ-2 (LHS)	3.9172	3.6769	1.7275	1.2960	4.9123	4.1093	4.0687	10.7004	9.2909	169.1815
IMQ-2 (LHS)	4.1892	3.8021	1.7909	1.3845	4.8665	3.9141	3.9273	9.9243	8.5706	136.4878
Linear (HS)	6.1278	6.0542	1.5412	1.1644	4.7204	4.3780	4.4194	4.5329	3.7792	55.2910
Cubic (HS)	5.3936	5.5210	2.0704	1.6000	6.9511	4.9966	5.0183	5.6291	4.6959	82.7652
Gauss (HS)	15.269	20.293	7.1762	5.6193	115.2787	4.1733	4.1727	5.3265	4.4659	**48.9555**
MQ (HS)	5.2290	5.6424	3.0226	2.3622	18.0381	4.4855	4.5286	4.6683	3.8869	62.8607
IMQ (HS)	4.8501	5.0505	2.4151	1.8799	10.6604	4.2210	4.2333	**4.3438**	**3.6220**	51.1284
TPS (HS)	5.4072	5.5296	1.6889	1.2851	4.6118	4.6630	4.6910	4.9165	4.0963	67.2636
Gauss-2 (HS)	4.7843	5.2010	1.7273	1.3218	4.5502	4.1880	4.1880	7.6788	6.5332	84.7080
MQ-2 (HS)	4.9174	5.0753	2.0255	1.5656	6.6438	4.4495	4.4672	4.4864	3.7317	58.5835
IMQ-2 (HS)	5.2947	5.3882	1.7069	1.3019	4.4239	4.1954	4.2004	5.4790	4.6158	54.0921
Linear (RS)	4.0657	3.4502	2.1022	1.7004	6.4889	4.0126	3.9669	10.3277	8.9190	148.5799
Cubic (RS)	3.2289	2.7843	1.7475	1.3601	4.0009	4.6060	4.5358	11.2713	9.7666	187.0106
Gauss (RS)	6.9129	5.8174	23.052	17.9454	1861.08	**3.8045**	3.8045	9.8617	8.5103	133.5786
MQ (RS)	3.4044	3.0009	2.1841	1.6952	6.5737	3.9907	4.0656	10.4481	9.0285	154.6638
IMQ (RS)	3.2568	2.8318	2.0606	1.6001	5.5758	3.8090	3.8185	10.0666	8.6880	139.9123
TPS (RS)	3.5139	2.7862	**1.3141**	**1.0169**	**2.6578**	4.3219	4.2296	10.7681	9.3128	165.8049
Gauss-2 (RS)	3.3497	3.2682	2.2501	1.7746	7.2793	3.8048	3.8048	9.8591	8.5082	133.5162
MQ-2 (RS)	**3.1444**	3.0117	1.6420	1.2693	3.7105	4.0568	4.0108	10.3911	8.9765	151.6385
IMQ-2 (RS)	3.3308	**2.7371**	1.5944	1.2601	4.0925	3.8119	**3.8043**	9.9933	8.6236	137.0649

**Table A4:** Ranking of various RBF metamodels using TOPSIS.

RBF Metamodel	LD Problem						HD Problem					
	First, Frequency		Second, Frequency		Third, Frequency		First, Frequency		Second, Frequency		Third, Frequency	
	CCi ^4^	Rank	CCi	Rank	CCi	Rank	CCi	Rank	CCi	Rank	CCi	Rank
Linear (LHS)	0.2003	27	0.3678	25	0.9568	15	0.5947	14	0.4680	15	0.3786	21
Cubic (LHS)	0.5723	16	0.7213	10	0.9767	6	0.8977	5	0.8652	6	0.0773	27
Gauss (LHS)	0.8362	4	0.4981	22	0.5098	26	0.0894	26	0.1522	26	0.4933	11
MQ (LHS)	0.7969	5	0.9427	1	0.9377	18	0.6659	12	0.5521	12	0.3240	24
IMQ (LHS)	0.7366	9	0.8965	2	0.9590	14	0.2664	20	0.2347	20	0.4607	17
TPS (LHS)	0.4229	22	0.6138	18	0.9677	10	0.8091	9	0.7169	8	0.2080	26
Gauss-2 (LHS)	0.6855	11	0.7678	9	0.9640	13	0.0894	27	0.1522	27	0.4935	10
MQ-2 (LHS)	0.6090	15	0.8039	5	0.9736	7	0.6320	13	0.5103	13	0.3510	22
IMQ-2 (LHS)	0.5010	20	0.6826	14	0.9675	11	0.1668	23	0.1836	23	0.4774	14
Linear (HS)	0.4072	24	0.5020	21	0.9160	23	0.9356	3	0.8952	3	0.9018	3
Cubic (HS)	0.7601	8	0.6963	11	0.9283	22	0.8796	6	0.8186	7	0.7611	8
Gauss (HS)	0.7697	7	0.6826	13	0.6257	25	0.5406	17	0.6330	11	0.9053	2
MQ (HS)	0.9157	1	0.8397	4	0.9147	24	0.9472	2	0.8978	2	0.8758	6
IMQ (HS)	0.8761	3	0.8527	3	0.9358	19	0.8543	7	0.8731	4	0.9317	1
TPS (HS)	0.6188	14	0.6449	17	0.9311	21	0.9306	4	0.8711	5	0.8430	7
Gauss-2 (HS)	0.7104	10	0.6854	12	0.9426	16	0.2753	19	0.3376	19	0.7094	9
MQ-2 (HS)	0.7756	6	0.7771	8	0.9400	17	0.9497	1	0.9044	1	0.8907	5
IMQ-2 (HS)	0.6362	13	0.6813	15	0.9342	20	0.6861	11	0.7155	9	0.8907	4
Linear (RS)	0.2128	26	0.1610	27	0.9660	12	0.5355	18	0.3474	18	0.4263	18
Cubic (RS)	0.4117	23	0.4563	23	0.9867	4	0.8479	8	0.6516	10	0.2562	25
Gauss (RS)	0.9059	2	0.7960	6	0.2621	27	0.1017	25	0.1573	24	0.4915	13
MQ (RS)	0.5617	18	0.7940	7	0.9729	8	0.5923	15	0.3853	16	0.4037	20
IMQ (RS)	0.5678	17	0.6676	16	0.9774	5	0.2622	21	0.2194	21	0.4664	16
TPS (RS)	0.3774	25	0.3318	26	0.9924	1	0.7157	10	0.4899	14	0.3496	23
Gauss-2 (RS)	0.6627	12	0.5901	19	0.9693	9	0.1017	24	0.1572	25	0.4918	12
MQ-2 (RS)	0.5004	21	0.5186	20	0.9889	3	0.5643	16	0.3642	17	0.4138	19
IMQ-2 (RS)	0.5180	19	0.4188	24	0.9903	2	0.1805	22	0.1906	22	0.4769	15

^4^ Closeness coefficient of *i*th metamodel.
